# Fucoxanthin Ameliorates Diabetic Kidney Disease via Modulating the Gut Microbiota and Tryptophan–Kynurenine Metabolic Pathway

**DOI:** 10.1155/jimr/1020462

**Published:** 2026-09-14

**Authors:** Donglin Guo, Jiayong Xie, Xueyun Dong, Hao Xu, Yunhan Xie, Xinyu Liu, Linlin Xu, Asmaa Ali, Min Chen, Leilei Zhang, Jiayuan He, Liang Wu, Keke Shao

**Affiliations:** ^1^ Clinical Laboratory of Shanxi Provincial People’s Hospital, Taiyuan 030012, China; ^2^ Nephrology Department, Xinghua People’s Hospital Affiliated to Yangzhou University, Taizhou 225700, China; ^3^ School of Medicine, Jiangsu University, Zhenjiang 212013, China, ujs.edu.cn; ^4^ Department of Laboratory Medicine, The First People’s Hospital of Yancheng, Yancheng 224000, China; ^5^ Department of Pulmonary Medicine, Abbassia Chest Hospital, EMOH, Cairo 11517, Egypt; ^6^ Public Experiment and Service Center, Jiangsu University, Zhenjiang 212013, China, ujs.edu.cn; ^7^ Zhenjiang Center for Disease Control and Prevention, Zhenjiang 212002, China; ^8^ Yancheng Clinical Medical College of Jiangsu University, Yancheng 224000, China

**Keywords:** diabetic kidney disease, fucoxanthin, gut microbiota, kynurenine, oxidative stress, tryptophan metabolism

## Abstract

This study employs a multiomics approach to investigate the protective effects of fucoxanthin (Fu) and explore its potential association with metabolic and signaling pathways in diabetic mice induced by a high‐fat diet combined with streptozotocin (STZ) administration. Diabetic model mice were randomly divided into normal control (NC), diabetic model, low‐dose Fu (50 mg/kg/day), and high‐dose Fu (100 mg/kg/day) groups. After 6 weeks of intervention, Fu treatment was associated with dose‐dependent improvements in glucose and lipid metabolism, reduced oxidative stress, decreased expression of inflammatory cytokines (tumor necrosis factor‐α [TNF‐α], interleukin‐6 [IL‐6], and IL‐1β) and renal fibrosis markers (TGF‐β1, α‐SMA, and Col1a1), along with notable attenuation of renal pathological damage. Exploratory analyses indicated that these phenotypic improvements were accompanied by reduced renal IDO1 expression, favorable shifts in tryptophan (Trp) metabolism, and upregulated AhR/CYP1A1 signaling. 16S rRNA sequencing revealed that Fu treatment was associated with a restoration of diabetes‐altered gut microbiota β‐diversity. Serum metabolomics identified that Fu administration correlated with alterations in multiple metabolites (including 5′‐S‐methyl‐5′‐thioadenosine, LPC 16:0, and bile acids), which are linked to key pathways such as Trp metabolism and bile acid biosynthesis. Network pharmacology predictions further suggested potential multitarget interactions of Fu with the PI3K‐Akt and AGE‐RAGE signaling pathways. Collectively, these findings indicate that Fu exerts potential renoprotective effects in diabetic mice, which are accompanied by a normalization of Trp–AhR pathway‐related metabolism and concurrent attenuation of inflammatory, oxidative, and fibrotic responses. While causal functional links remain to be fully elucidated, these observations provide a preliminary basis for further mechanistic investigation and support the potential of Fu as a dietary supplement or adjunctive strategy for diabetic kidney disease (DKD).

## 1. Introduction

Diabetic kidney disease (DKD) represents one of the most common and serious microvascular complications in diabetic patients. Approximately 30%–40% of individuals with diabetes ultimately progress to kidney failure, making DKD a leading cause of end‐stage renal disease (ESRD) [1]. Sustained hyperglycemia contributes to renal injury through multiple mechanisms, including the induction of inflammatory responses, oxidative stress, and metabolic disturbances, which collectively lead to glomerulosclerosis, tubulointerstitial fibrosis, and progressive loss of renal function [2]. Although current therapeutic strategies—such as strict glycemic control and the use of renin–angiotensin system inhibitors—partially slow disease progression, the incidence of DKD remains persistently high, and specific targeted therapies remain lacking [3]. Therefore, further elucidating the pathogenic mechanisms of DKD and developing novel renal protective agents are of considerable clinical importance [4].

Recent studies have identified chronic low‐grade inflammation and oxidative stress as central players in the onset and progression of DKD. Under hyperglycemic and dyslipidemic conditions, excessive accumulation of reactive oxygen species (ROS) induces mitochondrial dysfunction, activating signaling pathways such as NF‐κB and the NLRP3 inflammasome. This activation promotes the release of pro‐inflammatory cytokines (e.g., tumor necrosis factor‐α [TNF‐α], interleukin‐6 [IL‐6], and IL‐1β), thereby exacerbating glomerular basement membrane thickening and podocyte injury [5, 6]. Furthermore, the relationship between gut microbiota dysbiosis, disrupted metabolite profiles, and DKD is increasingly recognized [7]. Notably, recent evidence highlights the indoleamine 2,3‐dioxygenase 1 (IDO1)‐mediated tryptophan (Trp)–kynurenine (Kyn) pathway as a critical pathological axis. In the diabetic state, hyperactivation of this pathway leads to an excessive accumulation of Kyn, which acts as an endogenous ligand that chronically overactivates the aryl hydrocarbon receptor (AhR). This sustained AhR activation further incites renal inflammation, uncouples metabolic homeostasis, and accelerates fibrotic cascades, thereby serving as a pivotal molecular bridge linking gut dysbiosis to progressive kidney injury [8]. Therefore, targeting the regulation of the inflammation‐oxidative stress axis and the gut microbiota–Kyn pathway may provide new therapeutic avenues for DKD.

Fucoxanthin (Fu), a natural carotenoid derived from brown algae, exhibits remarkable antioxidant, anti‐inflammatory, and metabolic regulatory properties. Previous research has demonstrated that Fu can alleviate oxidative damage by activating the Nrf2/HO‐1 pathway, suppress inflammatory responses by inhibiting MAPK/NF‐κB signaling, and ameliorate metabolic disorders by modulating gut microbiota diversity [9]. In a diabetic model induced by a high‐fat diet combined with streptozotocin (STZ), Fu has been shown to improve insulin resistance and hepatic lipid accumulation; however, its protective effects against DKD and its regulatory role in the gut microbiota–Kyn axis remain unclear [10]. This study, employing a diabetic mouse model induced by a 60% high‐fat diet combined with low‐dose STZ, aims to investigate the effects of Fu intervention on renal inflammation, oxidative stress, gut microbiota structure, and the serum metabolic profile—particularly the Kyn pathway—thereby providing experimental evidence for its potential as a dietary supplement in DKD management.

## 2. Material and Methods

### 2.1. Establishment of Diabetic Mouse Model and Experimental Design

Eight‐week‐old male C57BL/6 mice were obtained from Phenok Biosciences (Shanghai) and maintained in SPF conditions at Jiangsu University’s animal facility [11]. Environmental parameters included controlled temperature (22 ± 2°C), humidity (50%–60%), and 12 h light/dark cycling with unrestricted access to food/water [11]. The study protocol received ethical approval from Jiangsu University (UJS‐IACUC‐AP‐2024030032).

Diabetes was induced through sequential HFD feeding (60% kcal fat) for 4 weeks, followed by three consecutive daily STZ injections (50 mg/kg, freshly prepared in citrate buffer, pH 4.5) [12]. 1 week post‐STZ administration, animals displaying fasting glucose >11.1 mmol/L were designated diabetic. Animals failing to meet this fasting glucose threshold were excluded from the study. The remaining eligible mice were allocated into study groups (*n* = 6) using simple randomization via a random number generator: healthy controls (standard diet), untreated diabetics (T2DM), and Fu‐treated diabetics receiving either 50 mg/kg/day (FuL) or 100 mg/kg/day (FuH) doses suspended in olive oil via gavage [13]. To ensure consistency, mice in the NC and untreated T2DM groups received equivalent volumes of the vehicle (olive oil) via daily gavage. Treatments commenced at week 7 and continued for 6 weeks until terminal collection under sevoflurane anesthesia. Biological samples were preserved appropriately (−80°C/fixed) following strict aseptic protocols. The detailed experimental procedures are provided in Supporting Information S1.

### 2.2. Serological and Hepatic Biochemical Analysis

Serum biochemical markers were quantified using standardized commercial kits (Nanjing Jiancheng Bioengineering Institute, China). Lipid profiles were examined by measuring total cholesterol (TC, #A111‐1‐1), triglycerides (TG, #A110‐1‐1), low‐density lipoprotein cholesterol (LDL‐C, #A113‐1‐1), and high‐density lipoprotein cholesterol (HDL‐C, #A112‐1‐1). Oxidative stress was evaluated via malondialdehyde (MDA, #A003‐1‐2), superoxide dismutase (SOD, #A001‐3‐2), glutathione peroxidase (GSH‐Px, #A005‐1‐2), catalase (CAT, #A007‐1‐1), and reduced glutathione (GSH, #A006‐2‐1). Inflammation‐related markers included TNF‐α (#H052‐1), IL‐6 (#H007‐1), and C‐reactive protein (CRP, #H206‐1‐1). Renal function was assessed by determining serum creatinine (Scr, #C011‐2‐1) and blood urea nitrogen (BUN, #C013‐2‐1), while fasting blood glucose (FBG) was measured using a glucometer.

For renal gene expression analysis, total RNA was isolated from ~50 mg kidney tissue using the Trizol reagent (Invitrogen). RNA quality was verified spectrophotometrically (Nanodrop 2000, Thermo Fisher Scientific), with A260/A280 ratios of 1.8–2.0 deemed suitable. cDNA was synthesized using HiScript II Q RT SuperMix (Vazyme, #R233‐01), followed by quantitative PCR amplification with AceQ SYBR Green Master Mix (Vazyme, #Q111‐02). β‐actin served as the endogenous control, and relative gene expression was calculated via the 2^−ΔΔCt^ method. The primer sequences for quantitative PCR are provided in Supporting Information Table S2.

For western blot analysis, total protein was isolated from kidney tissues using RIPA lysis buffer, and protein concentrations were quantified using a BCA assay. Equal amounts of protein were separated via SDS‐PAGE and transferred onto PVDF membranes. To minimize nonspecific background, membranes for total proteins (α‐SMA, AhR, p65, and β‐actin) were blocked with 5% nonfat milk, while those for the phosphorylated protein (pp65) were blocked with 5% bovine serum albumin (BSA) at room temperature for 1 h. Subsequently, the membranes were incubated overnight at 4°C with primary antibodies against α‐SMA (#14395‐1‐AP), AhR (#67785‐1‐Ig), pp65 (#80379‐2‐RR), p65 (#80979‐1‐RR), and β‐actin (#20536‐1‐AP), all purchased from Proteintech (Wuhan, China) and diluted at 1:1000. Then, the membranes were incubated with HRP‐conjugated goat anti‐rabbit secondary antibodies (Boster, Wuhan, China; #BA1054) at 1:15000 dilution for 1 h at room temperature. Protein bands were detected using an ECL kit and quantified by densitometry.

### 2.3. Renal Histopathological Analysis

Mouse kidney tissues were preserved in 4% paraformaldehyde overnight, sequentially dehydrated in graded ethanol, cleared in xylene, and paraffin‐embedded. Sections (4 μm) were prepared for histological evaluation using hematoxylin and eosin (H&E), Masson’s trichrome staining, and immunohistochemistry (IHC). For H&E analysis, deparaffinized slides were stained with hematoxylin (5 min), differentiated briefly in acid alcohol (10 s), rinsed thoroughly, and counterstained with eosin (30 s). After dehydration, the mounted slides were microscopically assessed to examine renal architecture and inflammatory responses. Masson’s trichrome staining employed sequential treatment with Weigert’s hematoxylin (5 min), Ponceau‐fuchsin (5 min), phosphomolybdic acid (2 min), and aniline blue (2 min) to quantify collagen deposition (blue‐stained fibers) as a marker of interstitial fibrosis. For IHC detection of RAGE (receptor for advanced glycation end products), endogenous peroxidase was quenched with 3% H_2_O_2_ (15 min), followed by antigen retrieval via microwave heating in citrate buffer (pH 6.0, 10 min). After blocking with 5% BSA (30 min), sections were incubated with a rabbit anti‐RAGE primary antibody (1:100, Proteintech #66521‐1‐Ig), probed with a HRP‐conjugated secondary antibody (37°C, 30 min), and developed with DAB. The RAGE expression was analyzed microscopically. To ensure quantitative rigor, all histological and immunohistochemical image analyses were performed by an investigator who was blinded to the experimental group assignments. For quantitative assessment, three randomly selected, nonoverlapping cortical fields per kidney section were captured under a microscope. The severity of pathological changes and the quantitative measurement of staining areas (such as the percentage of blue collagen deposition in Masson’s trichrome and the positive area for α‐SMA and RAGE in immunohistochemistry) were calculated using ImageJ software (NIH, Bethesda, MD, USA). Measurements were determined by calculating the percentage of the positively stained area relative to the total tissue area within each field based on predefined optical density thresholds.

### 2.4. Network Pharmacology Analysis of Fu in Diabetic Nephropathy (DN)

The molecular structure of Fu was acquired from PubChem, and its potential biological targets were predicted using computational tools including SEA, SwissTargetPrediction, SuperPred, and CBligand. Disease‐associated targets related to DN were identified through systematic searches of DisGeNET, OMIM, GeneCards, and TTD using “diabetic nephropathy” as the query. After eliminating duplicate entries, a consolidated DN target dataset was generated. Putative therapeutic targets of Fu against DN were determined by intersecting predicted drug targets with DN‐associated targets using bioinformatics tools.

These overlapping targets were then analyzed via the STRING database to establish a protein–protein interaction (PPI) network restricted to human proteins. The network was imported into Cytoscape 3.10.0 for graphical representation and topological analysis using the CytoNCA plugin. Targets exhibiting above‐median values for degree, betweenness, and closeness centrality were identified as hub targets.

Functional annotation and pathway enrichment analyses were conducted using DAVID, with Gene Ontology (GO) terms and KEGG pathways showing statistical significance (*p* < 0.05) considered biologically relevant. The most enriched pathways and GO terms were graphically represented as bubble plots. Finally, an integrated “compound‐target‐pathway” network was constructed in Cytoscape to elucidate Fu’s multitarget therapeutic potential against DN.

### 2.5. Fecal Microbiome Characterization Using 16S rRNA Gene Sequencing

For intestinal flora assessment, freshly collected stool specimens were immediately frozen at −80°C prior to DNA isolation using a commercial extraction kit (TIANGEN BIOTECH). Nucleic acid quality verification included spectrophotometric quantification (Nanodrop), fluorometric measurement (Qubit), and electrophoretic evaluation targeting fragment sizes exceeding 15 kb. Microbial community profiling focused on amplification of the V3‐V4 region (primers 341F/806R) followed by high‐throughput sequencing via Illumina NovaSeq 6000 with paired‐end 250 bp reads. Subsequent bioinformatics analysis utilized the QIIME2 framework, incorporating DADA2 algorithms for sequence refinement and ASV clustering. Taxonomic classification referenced both Silva and Greengenes reference databases, adopting a 97% sequence identity threshold. Microbial diversity metrics encompassed α‐diversity calculations (Shannon and Chao1 indices) and β‐diversity visualization through principal coordinates analysis employing Bray‐Curtis and weighted UniFrac measures. Distinctive bacterial taxa across experimental groups were determined through linear discriminant analysis effect size methodology applying significance thresholds of LDA > 2.0 and *p* < 0.05.

### 2.6. Targeted LC‐MS/MS Analysis for Renal Trp and Kyn

To specifically evaluate local metabolic disturbances in the kidney, targeted quantification of Trp and Kyn in the renal tissues was performed using LC‐MS/MS analysis. Briefly, frozen kidney tissue samples were homogenized in extraction solvent, centrifuged to precipitate proteins, and the supernatants were collected. The targeted metabolic profiling was conducted using an LC‐MS/MS system consisting of an ACQUITY UPLC I‐Class system coupled to a Xevo TQ‐S triple quadrupole mass spectrometer (Waters Corporation, Milford, MA, USA). Data acquisition and processing were performed using MassLynx software (Version 4.2, Waters Corporation). Metabolite concentrations were normalized to tissue weight and quantified using standard curves of authentic Trp and Kyn analytical standards.

### 2.7. Comprehensive Serum Metabolite Profiling Through UPLC‐QTOF‐MS Platform

In parallel to the targeted renal analysis, untargeted metabolic fingerprinting of serum specimens was conducted utilizing ultra‐high performance liquid chromatography‐quadrupole time‐of‐flight mass spectrometry (Waters Corporation). Sample pretreatment involved protein precipitation through methanol treatment, followed by centrifugal clarification. Chromatographic separation employed a reverse‐phase C18 column (2.1 × 100 mm, 1.7 μm particle size) with a binary mobile phase system comprising aqueous and organic modifiers (both containing 0.1% formic acid). Mass spectrometric detection simultaneously captured precursor and product ion spectra in dual polarity modes via the MSE acquisition protocol. Initial data refinement utilized the Progenesis QI platform executing feature detection (5 ppm mass accuracy threshold), chromatogram alignment, and intensity normalization procedures. To ensure system stability and data quality, a pooled quality control (QC) sample was prepared and injected every 10 samples throughout the analytical run. The injection order of all samples was randomized to minimize batch effects. Subsequent quality filtering eliminated inconsistent features (RSD > 30%) through XCMS processing. Multivariate pattern recognition incorporated PCA and OPLS‐DA modeling via SIMCA‐P software. To prevent model overfitting, the OPLS‐DA models were validated using a 200‐iteration permutation test. Metabolic discriminators were selected based on VIP scores exceeding unity (*p* < 0.05, FDR‐adjusted where possible). Compound annotation referenced major metabolomic repositories (HMDB/KEGG/METLIN) requiring a minimum spectral similarity of 60%, achieving Level 2 identification (putative annotation) according to the Metabolomics Standards Initiative (MSI) guidelines.

### 2.8. Statistical Evaluation

Numerical results are expressed as the mean ± SD and were analyzed using GraphPad Prism (v9.0). Distribution normality was examined via Shapiro–Wilk testing, while variance homogeneity was evaluated using Brown–Forsythe analysis. For normally distributed datasets with equal variance, intergroup comparisons were conducted through one‐way ANOVA followed by Tukey’s multiple comparison test when significant main effects (*p* < 0.05) were detected. Statistical significance was established at *p* < 0.05 for all hypothesis testing.

## 3. Results

### 3.1. Metabolic and Physiological Effects of Fu in Diabetic Mice

A high‐fat diet (60% kcal fat) coupled with STZ injections successfully induced diabetes mellitus (DM) in mice. Fu administration at low (FuL) and high doses (FuH) significantly modulated body weight dynamics, glycemic control, lipid metabolism, oxidative stress markers, and renal inflammation‐associated parameters.

Body weight progression differed notably between groups. Nondiabetic controls exhibited gradual weight gain, whereas DM mice displayed rapid initial weight gain (32.0 g peak at week 4), followed by progressive decline (lowest 26.0 g at week 12). Fu‐treated groups mirrored early DM weight trends but diverged after week 6, with FuH mice showing enhanced weight reduction. From these measurements, we inferred a dose‐responsive regulation (Figure 1A,B).

Figure 1Body weight and serum biochemical parameters in mice. (A) Body weight changes during the experimental period. (B) Final body weight at the end of the study. (C) Triglycerides (TG). (D) Total cholesterol (TC). (E) Low‐density lipoprotein cholesterol (LDL‐C). (F) High‐density lipoprotein cholesterol (HDL‐C). (G) Fasting blood glucose (FBG). (H) Serum creatinine (Scr). (I) Blood urea nitrogen (BUN). (J) Cystatin C (Cys‐C). (K) Malondialdehyde (MDA). (L) Superoxide dismutase (SOD). (M) Glutathione peroxidase (GSH‐Px). Data are expressed as mean ± SD (*n* = 6 mice per group). Statistical significance was determined using one‐way ANOVA followed by Tukey’s multiple comparison test. Exact *p*‐values for specific group comparisons (NC vs. DM, DM vs. FuL, and DM vs. FuH) are shown above the bars; “ns” denotes not significant.

**Figure:**
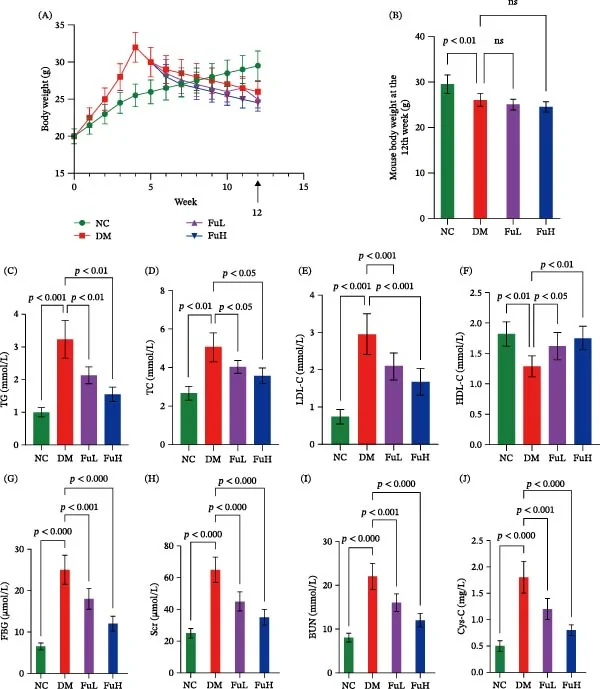

**Figure:**
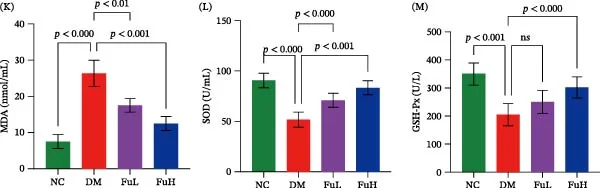

Lipid profiling revealed severe dyslipidemia in DM mice, including elevated TG, TC, and LDL‐C and depressed HDL‐C (*p* < 0.05). Fu intervention dose‐dependently ameliorated these lipid abnormalities, with FuH showing a more pronounced effect than FuL (Figure 1C–F). In addition, DM mice exhibited significantly elevated FBG, Scr, BUN, and Cys‐C levels, all of which were dose‐dependently reduced following Fu treatment (*p* < 0.05), with FuH producing the greater improvement (Figure 1G–J).

Oxidative imbalance in DM mice—marked by elevated MDA and diminished SOD/GSH‐Px activity (*p* < 0.05)—was counteracted by Fu treatment in a dose‐dependent manner. These observations infer an improvement in antioxidant capacity during diabetic pathology (Figure 1K–M).

### 3.2. Renal Protective Effects of Fu Against DKD

**Figure 2:**
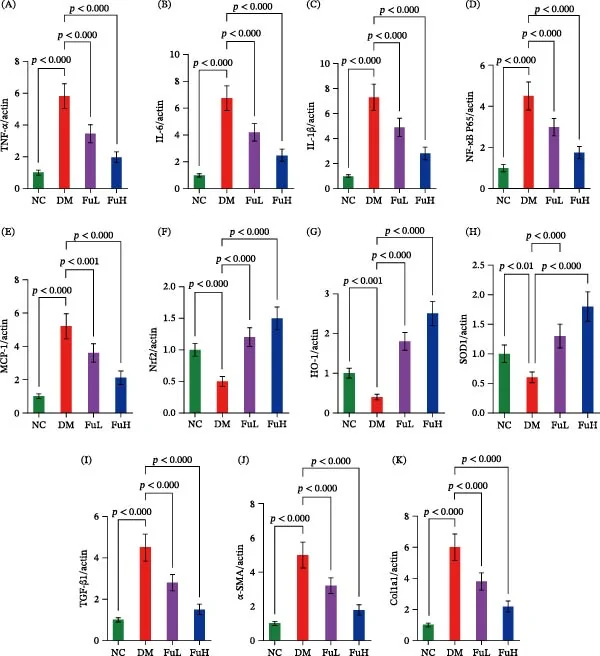
mRNA expression levels of inflammatory, oxidative stress, and fibrotic factors in mouse renal tissues determined by qPCR analysis. (A) TNF‐α, (B) IL‐6, (C) IL‐1β, (D) NF‐κB p65, (E) MCP‐1, (F) Nrf2, (G) HO‐1, (H) SOD1, (I) TGF‐β1, (J) α‐SMA, and (K) Col1a1. Data are expressed as mean ± SD (*n* = 6 mice per group). Statistical significance was evaluated using one‐way ANOVA followed by Tukey’s multiple comparison test. Exact *p*‐values for specific group comparisons (NC vs. DM, DM vs. FuL, and DM vs. FuH) are shown above the bars.

Quantitative PCR analysis demonstrated significant renal inflammatory activation in diabetic mice, with elevated TNF‐α, IL‐6, IL‐1β, NF‐κB p65, and MCP‐1 mRNA levels versus controls (*p* < 0.05). Fu administration dose‐dependently attenuated this cytokine upregulation, with FuH treatment restoring expression nearly to normal levels (*p* < 0.05). Oxidative stress markers exhibited parallel improvements, as evidenced by normalized Nrf2 pathway activity—the depressed Nrf2, HO‐1, and SOD1 expression in diabetic kidneys (*p* < 0.05) was progressively rescued by FuL and FuH treatments, with high‐dose intervention showing superior efficacy (*p* < 0.05) (Figure 2A–H).

**Figure 3:**
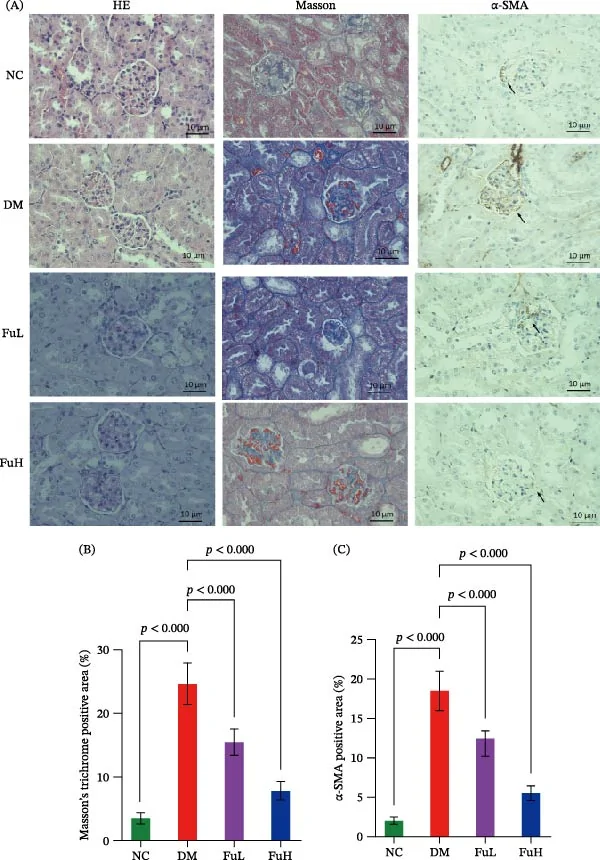
(A) Representative photomicrographs of mouse kidney sections showing H&E staining for general tissue morphology, Masson’s trichrome staining for collagen deposition (blue), and immunohistochemical staining for α‐SMA to evaluate myofibroblast activation (scale bar = 10 μm). Quantitative analysis of (B) Masson’s trichrome positive area (%) and (C) α‐SMA positive area (%). Images are representative of *n* = 3 mice per group. Data are expressed as mean ± SD. Statistical differences were determined using one‐way ANOVA followed by Tukey’s multiple comparison test. Exact *p*‐values are indicated above the bars.

Fibrosis‐related transcript analysis revealed dramatic diabetes‐induced upregulation of TGF‐β1, α‐SMA, and Col1a1 (*p* < 0.05), which was substantially mitigated by Fu treatment in a dose‐responsive pattern (Figure 2I–K). Histopathological assessment (H&E staining) corroborated the molecular data, showing that diabetic renal pathology—including glomerular hypertrophy, basement membrane thickening, tubular degeneration, and inflammatory infiltration—was markedly ameliorated by Fu intervention (Figure 3A).

To rigorously validate these antifibrotic effects, quantitative morphological analyses were performed. Masson’s trichrome staining revealed extensive collagen deposition in diabetic kidneys; quantitative evaluation demonstrated a striking increase in the collagen‐positive area in the DM group compared to normal controls (*p* < 0.05, Figure 3A, B). This fibrotic burden was significantly attenuated by Fu treatment in a dose‐dependent manner (*p* < 0.05 for both FuL and FuH), with FuH animals exhibiting near‐normal collagen distribution patterns. Crucially, immunohistochemical evaluation confirmed the transcriptomic findings at the protein level. The robust accumulation of α‐SMA‐positive myofibroblasts observed in diabetic kidneys was significantly reversed by Fu intervention, as evidenced by a profound reduction in the α‐SMA positive area (*p* < 0.05 for FuH vs. DM, Figure 3A, C).

### 3.3. Modulation of Renal Trp–Kyn Metabolism by Fu in DKD

**Figure 4:**
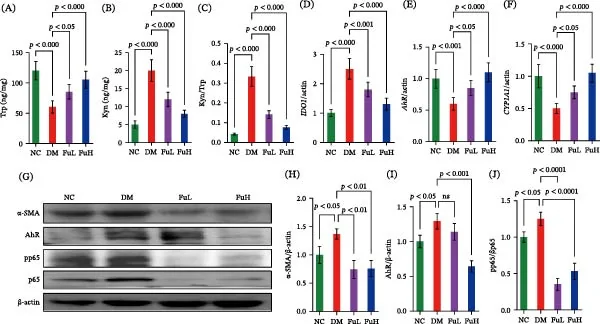
Renal tryptophan metabolic profile and associated gene and protein expression. The concentrations of (A) tryptophan (Trp) and (B) kynurenine (Kyn); (C) the Kyn/Trp ratio; and the relative mRNA expression levels of (D) the key enzyme *IDO1*, (E) its receptor *AhR*, and (F) its target gene *CYP1A1*. (G) Representative western blot bands and quantitative analysis of (H) α‐SMA, (I) AhR, and (J) pp65/p65 ratio in renal tissues, with β‐actin used as the internal control. Data are expressed as mean ± SD (*n* = 6 for A–F, and *n* = 3 for G–J). Statistical differences were determined using one‐way ANOVA followed by Tukey’s multiple comparison test. Exact *p*‐values for specific group comparisons (NC vs. DM, DM vs. FuL, and DM vs. FuH) are shown above the bars.

LC‐MS/MS and qPCR analyses revealed profound disturbances in renal Trp homeostasis and associated signaling pathways in diabetic mice (Figure 4A–F). The DM group showed a marked elevation of *IDO1* expression (*p* < 0.05, Figure 4D) accompanied by depleted Trp reserves (*p* < 0.05, Figure 4A) and accumulated Kyn metabolites (*p* < 0.05, Figure 4B). From this, we inferred heightened Trp catabolism, which was further confirmed by a dramatic increase in the Kyn/Trp ratio (*p* < 0.05, Figure 4C). Concurrent suppression of *AhR* (*p* < 0.05, Figure 4E) and *CYP1A1* (*p* < 0.05, Figure 4F) mRNA expression was measured, indicating a compromise in pathway activity under diabetic conditions.

Fu administration dose‐dependently normalized these metabolic perturbations. Both FuL and FuH treatments attenuated *IDO1* overexpression (*p* < 0.05) while reactivating *AhR* and *CYP1A1* transcription, with high‐dose intervention achieving expression levels comparable to normal controls. At the metabolite level, Fu treatment significantly restored Trp concentrations while reducing Kyn accumulation (*p* < 0.05), effectively normalizing the Kyn/Trp ratio in a dose‐responsive manner (Figure 4A–C).

To further investigate whether these metabolic shifts translate into alterations at the protein level and downstream pathological phenotypes, Western blot analysis was performed (Figure 4G–J). Intriguingly, while *AhR* mRNA was suppressed in the DM group, its total protein expression was significantly elevated (*p* < 0.05, Figure 4I), suggesting a potential posttranslational regulatory mechanism or compensatory feedback under diabetic stress. Notably, high‐dose Fu significantly suppressed this aberrant AhR protein accumulation (*p* < 0.05). Furthermore, the diabetic mice exhibited a marked increase in the pro‐fibrotic marker α‐SMA (*p* < 0.05, Figure 4H) and a heightened ratio of phosphorylated p65 to total p65 (pp65/p65) (*p* < 0.05, Figure 4J), indicating substantial renal fibrosis and NF‐κB inflammatory pathway activation. Fu intervention significantly down‐regulated α‐SMA protein expression (*p* < 0.05) and robustly inhibited p65 phosphorylation (*p* < 0.05), reversing these pathological trends.

These findings collectively demonstrate that Fu corrects diabetes‐induced dysregulation of renal Trp metabolism through simultaneous modulation of enzymatic activity (IDO1 suppression), metabolite balance restoration, and AhR pathway reactivation. Moreover, the synchronized mitigation of α‐SMA and pp65 levels strongly supports the hypothesis that AhR‐mediated mechanisms and downstream NF‐κB pathway inhibition contribute substantially to its anti‐inflammatory, antioxidant, and antifibrotic properties.

### 3.4. Exploratory Network Pharmacology Predictions of Fu’s Renoprotective Mechanisms

**Figure 5:**
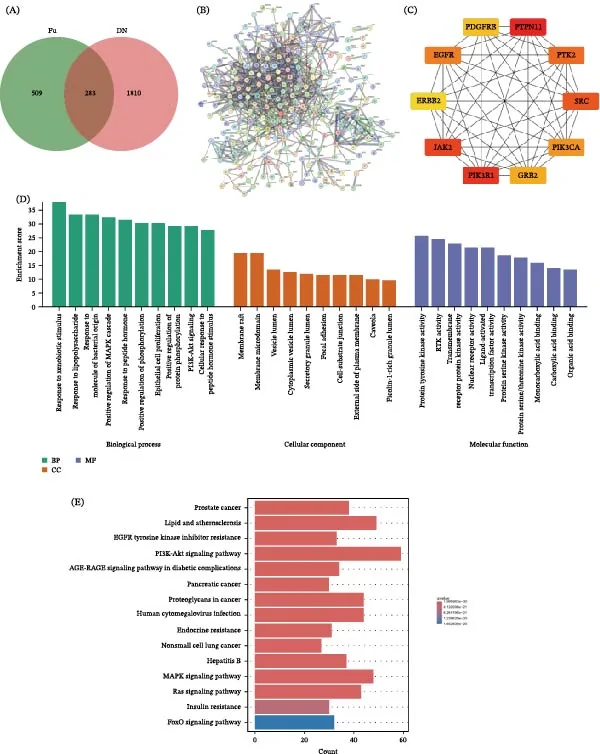
Network pharmacology analysis of fucoxanthin (Fu) for the treatment of diabetic nephropathy. (A) Venn diagram illustrating the overlap between Fu potential targets and diabetic nephropathy‐related targets. (B) Protein–protein interaction (PPI) network of the common targets. (C) Core targets identified from the PPI network. (D) Gene Ontology (GO) enrichment analysis. (E) Kyoto Encyclopedia of Genes and Genomes (KEGG) pathway enrichment analysis. Statistical significance for functional annotation and pathway enrichment was conducted using the DAVID database, with exact *p*‐values (*p* < 0.05) considered biologically relevant.

An integrated bioinformatic approach was used to explore potential multitarget actions of Fu against DKD. Database mining (SwissTargetPrediction/SuperPred) identified 792 candidate targets for Fu, while disease repositories (DisGeNET/GeneCards/OMIM) yielded 2093 DN‐associated proteins. Target interaction analysis revealed 283 overlapping candidates, which are inferred to be potential therapeutic nodes (Figure 5A). PPI network construction using STRING and Cytoscape uncovered key hub targets, including PTPN11, JAK2, PIK3R1, EGFR, and SRC (Figure 5B,C).

Functional annotation demonstrated significant enrichment in biological processes particularly involved in xenobiotic response (*p* < 0.05), MAPK cascade regulation (*p* < 0.05), and peptide hormone signaling (*p* < 0.05). Cellular localization analysis showed predominant association with membrane microdomains (*p* < 0.05) and vesicular compartments (*p* < 0.05), while molecular function categorization highlighted tyrosine kinase activities (*p* < 0.05) and nuclear receptor interactions (*p* < 0.05) (Figure 5D). Pathway mapping generated the hypothesis that Fu’s therapeutic effects may principally involve modulation of PI3K‐Akt signaling (*p* < 0.05), AGE‐RAGE axis in diabetic complications (*p* < 0.05), and insulin resistance pathways (*p* < 0.05) (Figure 5E).

### 3.5. Impact of Fu on Gut Microbial Ecology in DKD

**Figure 6:**
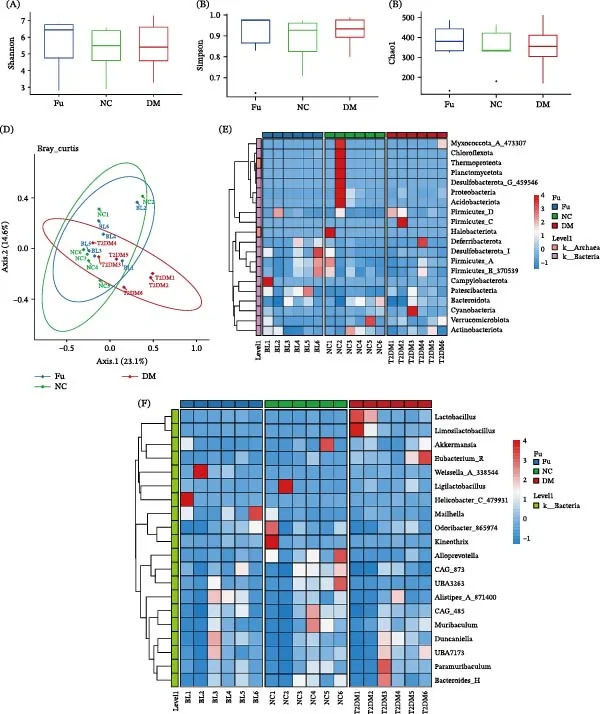
Effects of fucoxanthin on the gut microbiota in mice as determined by 16S rRNA sequencing. (A–C) Assessment of α‐diversity using the (A) Shannon, (B) Simpson, and (C) Chao1 indices. (D) β‐diversity analysis represented by a PCoA plot. Compositional profiles of the microbiota are displayed as heatmaps at the (E) phylum and (F) genus levels. Differential bacterial taxa among groups were determined via linear discriminant analysis effect size (LEfSe) with a threshold of LDA > 2.0 and *p* < 0.05.

Given that high‐dose Fu (FuH) demonstrated the most robust efficacy in mitigating metabolic disturbances and renal pathology in our preceding evaluations, we strategically prioritized this group to explore its influence on intestinal microbial communities using 16S ribosomal RNA sequencing. This approach aimed to maximize the identification of key microbial shifts associated with the strongest phenotypic response. Microbial α‐diversity metrics (Shannon, Simpson, Chao1) remained comparable across all groups, suggesting preserved ecological richness (Figure 6A–C). However, principal coordinate analysis (PCoA) of β‐diversity revealed distinct clustering patterns (PERMANOVA, T2DM vs. NC: pseudo‐*F* = 2.22, *p* < 0.024；T2DM vs. FU: pseudo‐*F* = 1.74, *p* < 0.043；NC vs. FU: pseudo‐*F* = 1.69, *p* < 0.046), with DM mice forming a separate group from both normal controls and FuH‐treated animals (Figure 6D), indicating an inferred FuH‐mediated restoration of diabetes‐altered microbiota composition.

Taxonomic profiling demonstrated FuH’s regulatory capacity across multiple bacterial lineages. Phylum‐level analysis showed that FuH significantly augmented Desulfobacterota populations while modulating Firmicutes subclades (Figure 6E). Genus‐specific evaluation identified FuH‐induced correction of diabetes‐associated dysbiosis markers: diminished Lactobacillaceae representatives were partially restored, while pathogenic genera *Mailhella* and *Alistipes_A_871400* showed reduced prevalence compared to untreated diabetic mice (Figure 6F). These microbiota alterations correlated with FuH’s systemic benefits, supporting the hypothesis that gut‐kidney axis involvement in its nephroprotective effects.

### 3.6. Metabolic Reprogramming Effects of Fu in Diabetic Serum Profiles

Principal component analysis of serum metabolites demonstrated distinct clustering patterns, revealing substantial metabolic perturbations induced by diabetic conditions compared to normal controls (Figure 7A). Notably,Fu administration resulted in a discernible shift away from the diabetic metabolic phenotype, inferring systemic metabolic modulation. This separation was further validated through orthogonal projection analysis (OPLS‐DA, Figure 7B). The robustness of the OPLS‐DA models was confirmed by 200‐iteration permutation testing, which indicated no overfitting (*R* ^2^ *Y* = 0.86–0.99 and *Q* ^2^ = 0.05–0.14, with a negative *Q* ^2^ intercept). Through comprehensive screening (VIP > 1, *p* < 0.05), we identified numerous dysregulated metabolites in both ionization modes (Figure 7C,D), including elevated phospholipids (LPC 16:0, LPC 23:0) and nucleotide derivatives alongside decreased bile acids (chenodeoxycholic acid), sphingolipids (S1P), and phenolic compounds.

Figure 7Analysis of serum metabolomic profile in mice. (A, B) Overview and supervised modeling of metabolic separations via PCA and OPLS‐DA score plots, respectively. (C, D) Identification of differential metabolites presented as heatmaps for ESI^+^ and ESI^−^ modes. (E, F) Summary of significantly enriched metabolic pathways derived from metabolites detected in ESI^+^ and ESI^−^ modes. Significant metabolic discriminators among the evaluated groups (NC, DM, and FuH) were selected based on variable importance in projection (VIP) scores > 1 and *p* < 0.05 (FDR‐adjusted where possible).

**Figure:**
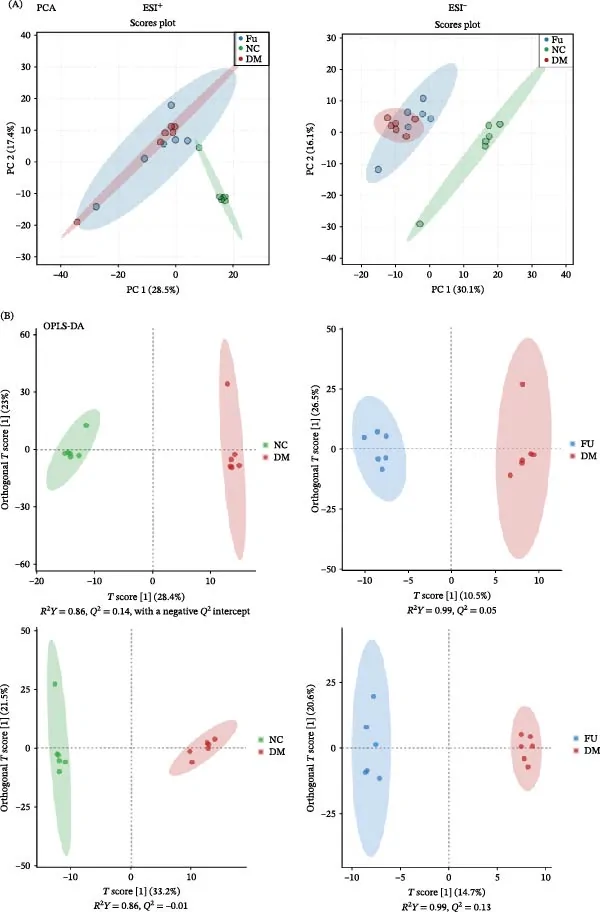

**Figure:**
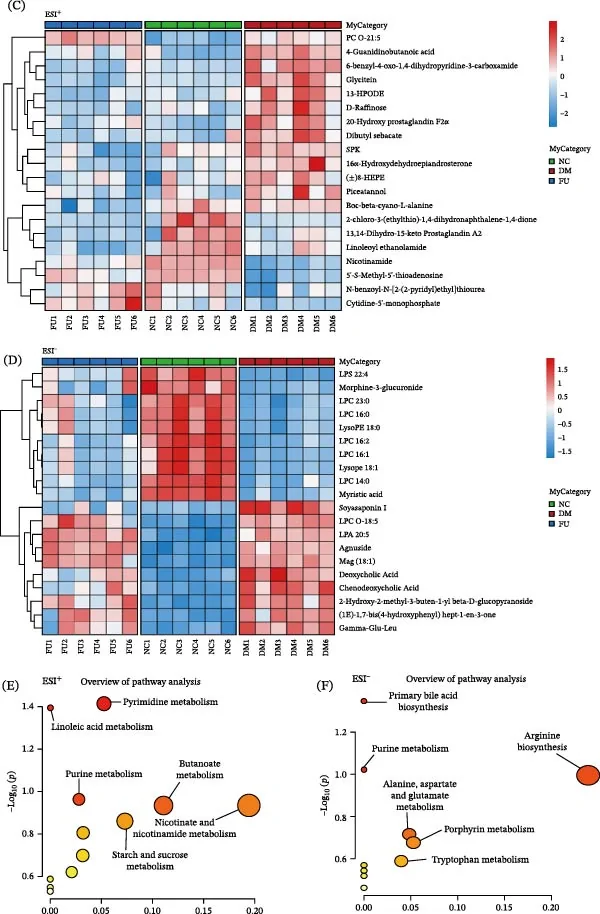

Metabolic pathway mapping revealed Fu’s involvement in multiple biochemical networks, particularly affecting nucleotide metabolism (pyrimidine/purine), lipid processing (linoleic acid pathway), amino acid utilization (Trp/arginine), and hepatic detoxification systems (bile acid biosynthesis) (Figure 7E,F). The observed metabolic shifts correlated with Fu’s established amelioration of diabetic complications, leaving it hypothetical that these circulating metabolites may serve as potential mediators of its therapeutic effects.

## 4. Discussion

This study systematically investigated the protective effects and underlying mechanisms of Fu against DKD using a mouse model induced by a high‐fat diet and low‐dose STZ. The results demonstrate that Fu not only significantly ameliorated hyperglycemia, dyslipidemia, and systemic oxidative stress but also markedly attenuated renal inflammation, fibrosis, and disruptions in Trp metabolism. Furthermore, integrated analyses employing network pharmacology, 16S rRNA sequencing of the gut microbiota, and untargeted serum metabolomics revealed that Fu treatment is associated with concurrent improvements across metabolic, microbial, and signaling parameters, suggesting a potential multitarget regulatory network.

First, Fu demonstrated significant metabolic regulatory effects in diabetic mice. High‐dose Fu effectively reduced FBG, TG, TC, and low‐density lipoprotein cholesterol (LDL‐C) levels while increasing high‐density lipoprotein cholesterol (HDL‐C), indicating its capacity to improve diabetes‐related metabolic disorders. Concurrently, Fu significantly enhanced the activities of SOD and GSH‐Px and reduced MDA content in serum, suggesting that it alleviates diabetes‐induced oxidative stress damage by bolstering the antioxidant defense system.

At the renal tissue level, this study revealed that Fu is associated with an interruption of the core pathological progression of DKD by synergistically inhibiting the inflammation‐oxidative stress axis. Under hyperglycemic conditions, the excessive accumulation of ROS in the kidney not only causes direct cellular damage but also acts as a potent second messenger, activating key inflammatory signaling hubs such as NF‐κB [14]. In the present study, the DM group showed upregulated expression of the NF‐κB p65 subunit in the kidney, accompanied by a burst release of downstream effector pro‐inflammatory cytokines like TNF‐α, IL‐6, and IL‐1β, creating a persistent inflammatory microenvironment. This chronic low‐grade inflammation is an initiating factor for podocyte injury, glomerular basement membrane thickening, and renal tubular epithelial cell transdifferentiation [15].

Importantly, a vicious cycle forms between inflammation and oxidative stress. On the one hand, activation of the NF‐κB pathway can further induce mitochondrial dysfunction, exacerbating ROS production. On the other hand, this study observed impaired function of the endogenous antioxidant defense system (e.g., the Nrf2/HO‐1 pathway) in the diabetic kidney, with significant suppression of Nrf2, HO‐1, and SOD1 expression, leading to a decreased capacity to scavenge oxygen free radicals and an inability to effectively counter escalating oxidative damage [16].

The synergistic exacerbation of inflammation and oxidative stress directly promotes the process of renal fibrosis. Pro‐inflammatory cytokines such as TNF‐α and IL‐1β have been shown to directly activate resident renal cells and stimulate them to secrete large amounts of key pro‐fibrotic factors, notably TGF‐β1 [17]. TGF‐β1 acts as a “master switch” for fibrosis. It drives the activation and proliferation of myofibroblasts (manifested as increased α‐SMA expression) via the Smad signaling pathway while potently stimulating the synthesis and deposition of extracellular matrix (particularly type I collagen, encoded by Col1a1) and simultaneously inhibiting its degradation, ultimately leading to tubulointerstitial fibrosis and glomerulosclerosis [18]. The extensive blue collagen deposition in the Masson staining and the significantly expanded α‐SMA‐positive areas in the immunohistochemistry of the DM group in this study are direct morphological manifestations of this series of molecular events.

Fu intervention appears to interrupt this vicious cycle at multiple points. Fu not only directly alleviates inflammation by upstream inhibition of NF‐κB activation but also significantly enhances the kidney’s antioxidant capacity by activating the Nrf2/HO‐1 pathway, thereby reducing ROS at the source, which drives inflammation and fibrosis [19, 20]. The combined anti‐inflammatory and antioxidant effects effectively curb the excessive activation of TGF‐β1 signaling, subsequently inhibiting myofibroblast transdifferentiation and abnormal collagen accumulation. This is intuitively corroborated by the nearly normal renal pathological structure observed in the high‐dose Fu group [21, 22]. Therefore, the protective effect of Fu against DKD is hypothesized to involve its coordinated regulation of the inflammation‐oxidative stress‐fibrosis network.

Fu treatment was associated with concurrent attenuation of inflammatory markers and restoration of antioxidant capacity, which coincided with reduced fibrotic indicators. These coordinated observations suggest that Fu may mitigate DKD by modulating the inflammation‐oxidative stress‐fibrosis network, although the precise directional relationships among these pathways remain to be functionally dissected.

Notably, this study highlights the regulatory role of Fu on the Trp–Kyn metabolic pathway in the diabetic kidney. Disrupted Trp metabolism has been established as a critical link connecting systemic metabolic abnormalities to local organ damage. In the diabetic state, as shown in our results, the expression of the enzyme indoleamine 2,3‐dioxygenase 1 (IDO1) is significantly upregulated in the renal tissue. This drives the aberrant metabolism of Trp via the Kyn pathway, leading to local Trp depletion and Kyn accumulation within the kidney. This metabolic imbalance has direct pathological significance: under chronic diabetic conditions, the continuous accumulation of Kyn contributes to a dysregulation of AhR signaling, leading to an altered transcriptional responsiveness that manifests as suppressed expression of AhR and its downstream target gene CYP1A1 [23, 24]. The AhR pathway plays a complex role in maintaining tissue homeostasis, and its impaired function in this tissue‐specific context can exacerbate inflammatory responses, oxidative stress, and promote fibrotic processes [25–27]. This study confirms that Fu intervention dose‐dependently reverses these abnormalities by inhibiting IDO1 overactivation, restoring the renal Trp/Kyn balance, and reactivating the AhR/CYP1A1 signaling axis. These coordinated alterations suggest that modulation of the Trp–Kyn–AhR pathway may represent a plausible but hypothetical mechanistic link contributing to Fu’s observed renoprotective profile.

In the current study, we focused on Trp metabolism and its downstream AhR signaling pathway and further analyzed the potential link between gut microbiota changes and these metabolic pathways. Analysis of the gut microbiota via 16S rRNA sequencing showed that Fu intervention significantly reversed diabetes‐induced gut microbiota dysbiosis. Beta‐diversity analysis indicated clear separation between the DM group and both the NC‐ and Fu‐treated groups, suggesting that Fu modulates the gut microecology [28]. At the phylum level, Fu treatment significantly increased the abundances of Desulfobacterota, Firmicutes_A, Firmicutes_B_370539, and Patescibacteria. At the genus level, the abundances of *Lactobacillus* and *Limosilactobacillus* decreased, while those of *Mailhella* and *Alistipes_A_871400* increased. These microbial shifts may indirectly influence the host metabolism and immune status [29, 30]. However, this study did not definitively identify specific bacterial taxa directly responsible for activating the Trp metabolism. For instance, Trp metabolism often involves bacterial production of indole derivatives, which can act as AhR ligands and regulate the host metabolism [31, 32]. However, in the present 16S rRNA sequencing results, the abundance changes of relevant bacteria (e.g., known Trp‐metabolizing bacteria) were not significant or were inconsistent with the expectations. This might be due to the limitations of the 16S rRNA technology [33, 34]. This technique, primarily based on sequencing the bacterial 16S rRNA gene, provides structural information about the microbiota but has a limited resolution. It cannot directly detect functional genes (e.g., genes for Trp‐metabolizing enzymes) or metabolic product activity, potentially missing key functional microbiota related to Trp metabolism [34, 35]. Furthermore, the impact of gut microbiota on Trp metabolism might occur through various indirect pathways, such as modulating inflammatory responses or gut barrier function, which were not fully explored in this experiment [36]. Future studies should employ metagenomic approaches to more precisely resolve functional changes in the gut microbiota, thereby fully elucidating the therapeutic potential of Fu in DKD.

Serum untargeted metabolomics revealed that Fu intervention significantly reversed systemic metabolic disorders in diabetic mice [37]. Specifically, levels of metabolites such as 5′‐S‐methyl‐5′‐thioadenosine (MTA) and LPC 16:0 were increased [38, 39], while levels of 4‐guanidinobutanoic acid, deoxycholic acid, and chenodeoxycholic acid were significantly decreased [40, 41]. These specifically altered metabolites are not isolated events; they collectively outline, from different angles, the complex metabolic regulatory network through which Fu may exert its renoprotective effects [42]. 5′‐S‐methyl‐5′‐thioadenosine (MTA) is a key intermediate in intracellular polyamine metabolism and methylation reactions [43]. It possesses potent anti‐inflammatory and antioxidant properties. Under diabetic and other stress conditions, MTA production is often impaired. The observed increase in serum MTA levels following Fu treatment suggests that Fu may promote the accumulation of this endogenous protective molecule, thereby inhibiting renal oxidative stress and inflammatory cascades, which aligns with the observed anti‐inflammatory and antioxidant effects of Fu [44]. The elevation of MTA might result from Fu’s direct or indirect regulation of the methylation metabolism or its mitigation of cellular damage.

LPC 16:0 (lysophosphatidylcholine 16:0) is an important product of phospholipid metabolism, and its biological function depends on both concentration and species. Unlike some long‐chain unsaturated LPCs implicated in inflammation, saturated LPCs like LPC 16:0 have been reported to be associated with the activation of the PPARγ signaling pathway [45]. PPARγ agonists are known to have anti‐inflammatory, anti‐fibrotic, and insulin‐sensitizing effects in DKD [46]. Therefore, the rebound in LPC 16:0 levels might indicate that Fu, by modulating phospholipid metabolism, activates the protective renal PPARγ pathway, thereby contributing synergistically to its renoprotective effects [47].

4‐Guanidinobutanoic acid is a byproduct of arginine metabolism that can compete with nitric oxide synthase (NOS) for the substrate L‐arginine [48], leading to reduced nitric oxide (NO) production and increased generation of ROS [49]. NO is crucial for maintaining renal blood flow and endothelial function [50]. The accumulation of 4‐guanidinobutanoic acid in diabetes exacerbates endothelial dysfunction and oxidative stress. The significant reduction in its serum concentration by Fu implies a normalization of the L‐arginine metabolic pathway, potentially helping to restore renal microvascular function and alleviate oxidative damage [51].

Deoxycholic acid and chenodeoxycholic acid are primary bile acids. The finding that these primary bile acids were elevated in the serum of diabetic mice and decreased after Fu intervention is highly significant. Bile acids are not merely fat emulsifiers but also important signaling molecules that regulate energy metabolism and inflammation by activating receptors like the farnesoid X receptor (FXR). In the context of diabetes, excessive bile acids, particularly hydrophobic primary bile acids, can induce bile acid toxicity and oxidative stress in the liver and kidneys. Their reduction strongly suggests that Fu might act through two interrelated mechanisms: first, Fu, potentially by ameliorating gut microbiota dysbiosis (as indicated by the changes in Desulfobacterota, etc., mentioned earlier) [52], reduces intestinal bile acid reabsorption, promotes their excretion, and thereby lowers circulating bile acid levels [53]. Second, the reduced bile acid levels lessen their direct toxicity to renal cells and may indirectly influence systemic inflammatory and metabolic status by modulating FXR signaling [54, 55].

Collectively, the concurrent improvements in gut microbiota composition, serum metabolite profiles, renal Trp–Kyn/AhR signaling, and inflammatory‐fibrotic markers following Fu treatment establish a strong correlative framework linking these biological layers. While these integrated multiomics observations strongly suggest that Fu’s renoprotective effects may be associated with modulation of the gut microbiota–Trp–Kyn–AhR axis, it is important to explicitly acknowledge that the present study design does not permit definitive causal inference, and this highly integrated mechanistic model remains largely hypothetical. The associations identified herein should be interpreted as hypothesis‐generating evidence that warrants rigorous validation through targeted interventional approaches (e.g., fecal microbiota transplantation, antibiotic depletion, or pharmacological/genetic modulation of AhR/IDO1) in future studies.

In summary, the alterations in these differential metabolites collectively indicate that Fu hypothetically confers renoprotection through synergistic, multitargeted mechanisms. It correlates with a rectification of the core disturbance in the Trp–AhR pathway and parallels the systemic ameliorates of diabetes‐induced metabolic dysregulation through multiple complementary approaches. These include elevating endogenous protective molecules such as MTA, activating beneficial signaling pathways like the LPC 16:0‐PPARγ axis, and reducing the accumulation of harmful metabolites, including 4‐guanidinobutanoic acid and specific bile acids. Furthermore, substantial crosstalk exists between bile acid metabolism, the gut microbiota, and Trp metabolism. The remodeling of the gut microbiota by Fu may contribute to the reduction in serum bile acid levels and could potentially facilitate AhR pathway activation by modifying the production of microbial AhR ligands, such as indole derivatives [56]. Consequently, the observed therapeutic profile of Fu against DKD appears closely associated with an integrated regulatory process involving the “gut‐metabolism‐immune” axis, although several of these proposed mechanistic links remain to be definitively proven. Within this observational framework, the modulation of Trp metabolism and AhR pathway activity emerges as a closely linked node, while parallel shifts in other metabolic pathways may provide complementary supportive effects [57].

Exploratory network pharmacology analysis predicted potential multitarget mechanisms of Fu against DKD. Core predicted targets included PTPN11, JAK2, PIK3R1, EGFR, etc., involving several signaling pathways closely related to diabetic complications, such as PI3K‐Akt, AGE‐RAGE, and MAPK. These results provide a theoretical basis for further elucidating the molecular mechanisms of Fu.

Although this study systematically revealed the multifaceted protective effects of Fu against DKD and its association with Trp metabolism and the gut microbiota, some limitations remain. First, while we observed that Fu remodels gut microbiota structure and activates the renal AhR signaling pathway, the 16S rRNA sequencing technology failed to definitively identify key bacterial genera directly responsible for Trp metabolism and producing AhR ligands. This lack of direct evidence leaves the causal link within the “gut microbiota‐AhR” axis unconfirmed [58, 59]. Second, although we confirmed AhR pathway activation at the gene expression and metabolite levels, we did not directly validate in vivo whether AhR activation is necessary for Fu’s renoprotective effects using interventions like specific AhR antagonists. The central role of this pathway in Fu’s efficacy awaits further confirmation via gain‐ and loss‐of‐function experiments [60]. Finally, the multiple potential targets and pathways predicted by network pharmacology and metabolomics (e.g., PI3K‐Akt, bile acid‐FXR axis) have not been validated or functionally dissected at the protein level in the renal tissue; therefore, these specific findings remain exploratory and speculative, requiring targeted experimental validation in future studies. Fourth, direct urinary functional readouts such as albumin excretion, albumin‐to‐creatinine ratio, or creatinine clearance were not included as urine samples were not prospectively collected or preserved during the intervention period. While serum creatinine and BUN, combined with comprehensive histopathology and molecular fibrosis/inflammation markers, provide a well‐validated preclinical framework for assessing renal injury, the absence of serial urinary indices limits the direct clinical translation of our functional findings. Fifth, the sample size of *n* = 6 per group, while sufficient for primary biochemical and histological endpoints, is relatively small for microbiome and untargeted metabolomics analyses. Future studies with larger cohorts and a priori power calculations are necessary to validate these omics‐based conclusions. Sixth, while our untargeted serum metabolomics analysis provided robust putative annotations (MSI Level 2) through spectral database matching, these differential metabolites (such as MTA and LPC 16:0) were not further confirmed using authentic analytical standards. Future targeted absolute quantification using reference standards is required to validate these metabolic biomarker candidates. These aspects require further investigation in subsequent studies [61].

## 5. Conclusion

This study demonstrates that Fu effectively alleviates kidney injury in mice induced by a high‐fat diet combined with STZ. Its protective effects are associated with the multiple targets and pathways. Building on improvements in glucolipid metabolic disorders and systemic oxidative stress, Fu treatment correlates with an attenuation of the core pathological axis of “inflammation‐oxidative stress‐fibrosis” by suppressing NF‐κB‐mediated renal inflammation, activating the Nrf2 antioxidant defense, and blocking the TGF‐β1/Smad pro‐fibrotic signaling. Concurrently, Fu is associated with the normalization of systemic and renal local Trp metabolic disturbances by inhibiting IDO1 overactivation, restoring the Trp/Kyn balance, and concurrently activating the crucial homeostatic regulatory pathway of the renal AhR/CYP1A1. Furthermore, the study found that Fu significantly ameliorates diabetes‐associated gut microbiota dysbiosis and serum metabolomic disorders. The alterations in pathways such as bile acid and phospholipid metabolism, alongside improved Trp metabolism, suggest a coordinated metabolic response. Furthermore, our 16S rRNA analysis reveals that Fu partially restores diabetes‐associated gut microbiota dysbiosis at the taxonomic level. While functional metagenomic validation is still required, we hypothesize that Fu’s renoprotective effects may involve an interplay between metabolic regulation, immune modulation, and potential gut microecological shifts. Overall, Fu was associated with beneficial changes in metabolic, inflammatory, microbial, and metabolomic parameters in this model, while the causal hierarchy among these processes remains to be clarified. This provides a strong foundation for future mechanistic studies into its use as an adjunctive strategy and coordinately regulating multiple processes, including the Trp–AhR pathway, inflammation, oxidative stress, and fibrosis. This provides a solid experimental basis for its potential use as a dietary supplement or an adjunctive therapeutic strategy.

**Nomenclature**
TNF‐α: Tumor necrosis factor‐alpha
IL‐6: Interleukin‐6
IL‐1β: Interleukin‐1 beta
TGF‐β1: Transforming growth factor‐beta 1
α‐SMA: Alpha‐smooth muscle actin
Col1a1: Collagen type I alpha 1
IDO1: Indoleamine 2,3‐dioxygenase 1
AhR: Aryl hydrocarbon receptor
CYP1A1: Cytochrome P450 family 1 subfamily A member 1
PI3K‐Akt: Phosphoinositide 3‐kinase/protein kinase B
AGE‐RAGE: Advanced glycation end products and receptor for advanced glycation end products
NF‐κB: Nuclear factor kappa B
NLRP3: NLR family pyrin domain containing 3
Nrf2: Nuclear factor erythroid 2‐related factor 2
HO‐1: Heme oxygenase‐1
MAPK: Mitogen‐activated protein kinase.

## Author Contributions

Donglin Guo and Jiayong Xie designed the experiments. Donglin Guo, Jiayong Xie, and Xueyun Dong carried out the experiments. Hao Xu, Yunhan Xie, Xinyu Liu, and Linlin Xu analyzed the 16S rRNA sequencing data. Asmaa Ali, Min Chen, Leilei Zhang, and Jiayuan He analyzed the LC‐MS/MS data. Donglin Guo, Jiayong Xie, Xueyun Dong, Asmaa Ali, Liang Wu, and Keke Shao wrote the manuscript.

## Funding

This research was funded by the Shanxi Provincial Basic Research Program—Young Scientists Research Project (Grant 202403021222415), the Taizhou Science and Technology Support Program (Social Development) Project (Grant SSF20230126), the Scientific Research Project of Yancheng Municipal Health Commission (Grants YK2024116 and YK2024120), the Jiangsu University 2023 Medical Education Collaborative Innovation Fund Project (Grant JDYY2023107).

## Conflicts of Interest

The authors declare no conflicts of interest.

## Supporting Information

Additional supporting information can be found online in the Supporting Information section.

## Supporting information


**Supporting Information** S1: Animal modeling and experimental design. Table S2: Genes and primer sequences analyzed by qPCR.

## Data Availability

The data that support the findings of this study are available from the corresponding author upon reasonable request.
