# Supplementary material for: Fucoxanthin Ameliorates Diabetic Kidney Disease via Modulating the Gut Microbiota and Tryptophan–Kynurenine Metabolic Pathway
Source: J Immunol Res. 2026 Sep 14;2026:1020462. doi: 10.1155/jimr/1020462 (PMC13575650; doi:10.1155/jimr/1020462)
Supplement: Supplementary file 1 [file JIMR-2026-1020462-s001.docx]

**Supplement**

*S1. Animal Modeling and Experimental Design.* Male C57BL/6 mice (8 weeks old) were supplied by Phenok Biosciences (Shanghai, China; Production License No.: SCXK(Hu)2023-0008) and maintained under specific pathogen-free (SPF) conditions in the Laboratory Animal Center of Jiangsu University. The housing environment was controlled at 22 ± 2°C, 50–60% humidity, with a 12 h light/dark cycle. All animals had free access to food and water. The experimental procedures were approved by the Institutional Animal Care and Use Committee of Jiangsu University (Approval No.: UJS-IACUC-AP-2024030032).

A type 2 diabetic mouse model was established by feeding a high-fat diet (HFD; 60% kcal from fat, Nanjing Synergy Scientific Co., Ltd., China) for 4 weeks, followed by intraperitoneal injection of a low-dose of streptozotocin (STZ; Sigma-Aldrich, USA). The STZ was freshly dissolved in 0.1 M sodium citrate buffer (pH 4.5) and administered at 50 mg/kg for three consecutive days. One week after the last STZ injection, mice with fasting blood glucose levels ≥ 11.1 mmol/L were considered diabetic and included in the study.

The mice were randomly divided into four groups (n = 6 per group): normal control (NC) fed a standard diet, diabetic model (T2DM), fucoxanthin low-dose (FuL, 50 mg/kg/day), and fucoxanthin high-dose (FuH, 100 mg/kg/day). Fucoxanthin (Fu, ≥95% purity, Sigma-Aldrich, USA) was dissolved in olive oil. Starting from the 7th week, the FuL and FuH groups received Fu *via* oral gavage daily for 6 weeks, while the NC and T2DM groups received an equal volume of vehicle.

The total experimental duration was 12 weeks. At the end of the study, mice were anesthetized with sevoflurane (3–4% for induction, 1.5–2% for maintenance; RWD Life Science, China) and euthanized. Serum, kidney tissues, and colonic contents were collected for subsequent analyses. All samples were stored at –80°C or fixed appropriately to ensure data reliability. All procedures were performed under aseptic conditions in compliance with animal ethics guidelines.

Table S2 Genes and Primer Sequences Analyzed by qPCR

| Gene | Forward primer （5'→3'） | Reverse primer（5'→3'） |
| --- | --- | --- |
| Nrf2 | CTGAACTCCTGGACGGGAC | CGGTGGGTCTCCGTAAATG |
| HO-1 | AAGCCGAGAATGCTGAGTTCA | GCCGTGTAGATATGGTACAAGGA |
| SOD1 | TGGAGACCTGGGCAATGTC | TTTCCACCTTTGCCCAAGTC |
| TNF-α | CAGGCGGTGCCTATGTCTC | CGATCACCCCGAAGTTCAGT |
| IL-1β | GCAACTGTTCCTGAACTCAACT | ATCTTTTGGGGTCCGTCAACT |
| MCP-1 | TTAAAAACCTGGATCGGAACCAA | GCATTAGCTTCAGATTTACGGGT |
| TGF-β1 | CTCCCGTGGCTTCTAGTGC | GCCTTAGTTTGGACAGGATCTG |
| α-SMA | TGCTGACAGAGGCACCACTG | GCTGGAAGGTGGACAGCGAG |
| IDO1 | ATGGGCGTATGTGTGAAATAG | GCAAAGCGTCATCTTCACAC |
| AhR | TCTGGGACATCACCTACGAG | GTTGTCCCATGCAGAGGTC |
| CYP1A1 | TCTTCACCCGAGCTTCAG | CAGGAATCCTTGTGCTCC |
| β-actin | GGCTGTATTCCCCTCCATCG | CCAGTTGGTAACAATGCCATGT |
